# Li-Ion Conductive Li_1.3_Al_0.3_Ti_1.7_(PO_4_)_3_ (LATP) Solid Electrolyte Prepared by Cold Sintering Process with Various Sintering Additives

**DOI:** 10.3390/nano12183178

**Published:** 2022-09-13

**Authors:** Mykola Vinnichenko, Katja Waetzig, Alf Aurich, Christoph Baumgaertner, Mathias Herrmann, Chang Won Ho, Mihails Kusnezoff, Chang Woo Lee

**Affiliations:** 1 Fraunhofer IKTS, Winterbergstr. 28, 01227 Dresden, Germany; 2 Department of Chemical Engineering (Integrated Engineering), College of Engineering, Kyung Hee University, 1732, Deogyeong-daero, Giheung, Yongin 17104, Gyeonggi, Korea

**Keywords:** solid-state electrolyte, densification, cold sintering process, ionic conductivity

## Abstract

The density, microstructure, and ionic conductivity of solid electrolyte Li_1.3_Al_0.3_Ti_1.7_(PO_4_)_3_ (LATP) ceramics prepared by cold sintering using liquid and solid sintering additives are studied. The effects of both liquid (water and water solutions of acetic acid and lithium hydroxide) and solid (lithium acetate) additives on densification are investigated. The properties of cold-sintered LATP are compared to those of conventionally sintered LATP. The materials cold-sintered at temperatures 140–280 °C and pressures 510–600 MPa show relative density in the range of 90–98% of LATP’s theoretical value, comparable or higher than the density of conventionally sintered ceramics. With the relative density of 94%, a total ionic conductivity of 1.26 × 10^−5^ S/cm (room temperature) is achieved by cold sintering at the temperature of 200 °C and uniaxial pressure of 510 MPa using water as additive. The lower ionic conductivities of the cold-sintered ceramics compared to those prepared by conventional sintering are attributed to the formation of amorphous secondary phases in the intergranular regions depending on the type of additives used and on the processing conditions selected.

## 1. Introduction

Solid-state Li-ion batteries are drawing increasing attention due to their inherent safety since they do not use flammable liquid electrolytes typical of conventional Li-ion cell technology [1]. Additionally, they are expected to have higher lifetime compared to conventional ones due to lower reactivity of solids compared with liquids at the same temperature. Oxide-based solid electrolytes such as Li_1.3_Al_0.3_Ti_1.7_(PO_4_)_3_ (LATP) are the most promising solid Li-conductors because they can be fabricated in required quantities under ambient conditions [2] and show high Li-ion conductivity approaching at room temperature 5 × 10^−3^ S/cm for single crystals [3] and ≤10^−3^ S/cm for sintered ceramics [4]. In order to achieve high ionic conductivities, LATP requires conventional sintering at temperatures within the range 800–1100 °C. Such high temperatures lead to Li_2_O vaporization, formation of non-conductive secondary phases, and formation of cracks by anisotropic grain growth [5,6]. Moreover, this hinders co-sintering with cathode material [7].

In order to address these problems, novel sintering approaches are required for manufacturing of the Li-ion solid electrolytes and cells based thereof. Cold sintering is a new processing method that enables densification of ceramic materials comparable to the case of conventional sintering but at temperatures well below 400 °C and with short holding times of 1 h or less [8]. Although not all mechanisms of cold sintering have been elucidated, it is presently assumed that the observed densification of ceramic materials is enabled by a transient liquid phase, which facilitates the mass transport through the grain boundaries and accelerates surface diffusion, dissolution–precipitation processes, and plastic deformation [9]. In contrast to conventional sintering, high temperatures (>1000 °C) are not required to initiate the necessary mass transport processes. Pressure in the range of a few hundreds of megapascals and liquid additives enable the mass transport and thus the material densification at low temperatures. These key process differences are supposed to allow sintering of ceramics and composites that are thermodynamically unstable at high temperatures without changing their stoichiometry and phase composition. Due to the semi-open character of the sintering press chamber, cold sintering is expected to enable different mass transport compared to a conventional hydrothermal process [10].

These unique characteristics make cold sintering relevant for a wide range of materials and their combinations [11], leading to its growing importance especially for development of solid-state batteries using LATP as a solid electrolyte. Based on this approach, LATP ceramics with relative densities as high as 90% after sintering at temperatures as low as 120–130 °C were recently demonstrated [12,13]. Cold sintering at the temperature of 120 °C and the pressure of 420 MPa using deionized water with *N*-Methyl-2-pyrrolidone (NMP) as additive followed by post-annealing at 650 °C yielded LATP samples with relative densities of 80% and ionic conductivities of 2.8 × 10^−5^ S/cm [12]. Combining acetic acid with NMP and the same thermal treatment and pressure allowed reaching relative densities of 93% and ionic conductivities of 8 × 10^−5^ S/cm. This result points to the importance of ensuring sufficient mass transport during sintering, which is realized in that case by using acetic acid as a sintering aid. A different approach to reduce the sintering temperature was based upon adding water solutions of bis(trifluoromethanesulfonyl)imide (LiTFSI) Li-salts [13]. These LATP-LiTFSI composite electrolytes showed a relative density of 90% and ionic conductivity of 2.3 × 10^−4^ S/cm directly after the cold sintering at a temperature of 130 °C and a pressure of 620 MPa. Although the exact sintering mechanism leading to this high-performance material is not clear, it is assumed that the Li-salt in concentrations 5–22 vol.% improves the contact among the LATP grains already at substantially reduced temperatures. Due to the known toxicity of the salt and due to possibility of the LATP-independent Li-ion conduction through the salt, further research is necessary to establish practical approaches to the cold sintering of LATP.

The literature results show that selection of sintering additives and cold sintering process optimization are of critical importance to achieve high ionic conductivity in resulting LATP ceramics. Therefore, in this study, the microstructure, phase composition, density, and ionic conductivity of the LATP solid electrolytes are investigated along with their dependence on different types of sintering additives and varied sintering temperatures during the cold sintering process, focusing on non-toxic additives. The studies are accompanied by thermogravimetric analysis of the material being sintered with emission gas mass spectrometry.

## 2. Materials and Methods

### 2.1. Pre-sintering Powder Preparation

LATP powder with mean particle size of d_50_~0.8 µm and phase purity > 96% was prepared using the sol–gel route and subsequent thermal treatment and milling [4]. Before preparing the sample for cold sintering, the powder was additionally de-agglomerated in a ball mill (Pulverisette 7 premium line, Fritsch GmbH, Idar-Oberstein, Germany) with zirconia balls (Ø 3 mm) in ethanol. Then, the powder was homogeneously mixed with 20 wt.% of liquid additives using mortar and pestle. As liquid additives, deionized water (H_2_O) as well as 1.66 M aqueous solution of acetic acid (HAc, 100 % acetic acid, Merck, Darmstadt, Germany) and 0.1 M aqueous solution of lithium hydroxide (LiOH, LiOH·H_2_O, Sigma Aldrich, Taufkirchen, Germany) were used. In addition, the experiment with solid additive was performed using dry milling of the LATP powder along with 3.2 wt.% of lithium-acetate dihydrate (LiAc, LiAc·2H_2_O, Alfa Aeasar, Karlsruhe, Germany, melting point of the water-free LiAc is 280–285 °C) in the ball mill to obtain a homogenized mixture. The corresponding milling parameters were chosen very carefully to prevent possible heat generation within the grinding beads and thus melting of the salt.

The LATP powder from the same batch was also used for conventional sintering to provide reference data for comparison as described in the following sub-section.

### 2.2. Cold Sintering

**Figure 1:**
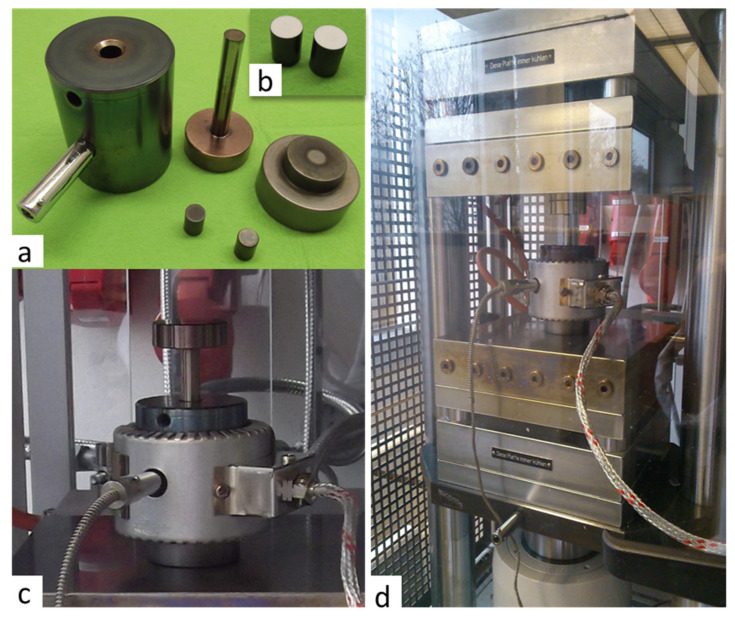
Experimental setup used for cold sintering: (**a**) pellet die 10 mm diameter; (**b**) dies with h-BN anti-adhesion coatings; (**c**) heating band around the pellet die; (**d**) electro-hydraulic 2-column laboratory press with mounted pellet die and heating system.

The freshly prepared mixture of the LATP powder with sintering additives was immediately transferred to the cold sintering facility and placed into die with a diameter of 10 mm. Prior to each experiment, the dies were spray-coated with antiadhesive material based upon hexagonal BN (h-BN). Then, the LATP was treated at high pressure using electro-hydraulic 2-column laboratory press (PW 40 EH-PRESSYS, P/O/Weber GmbH, Remshalden, Germany) with heating-cooling plate system (HKP 500, P/O/Weber GmbH). To ensure a uniform temperature distribution during sintering, the pellet die was covered with a band heater (Figure 1).

The temperature and uniaxial pressure during cold sintering were varied in the range of 140–280 °C and 510–600 MPa, respectively. Table 1 provides an overview of the temperatures, pressures, and dwell time applied in the sintering experiments. In case of a peak temperature of 140 °C, the sintering was performed at the pressure of 600 MPa for 1 h. The heating rate to 140 °C was of 13.5 K/min. For the peak temperatures of 160 and 180 °C, the modified heating profile was used: the sample was first ramped from RT to 140 °C at 13.5 K/min and held for 0.5 h, then it was further ramped to the peak temperature at 15 K/min and held for 1 h at this temperature keeping the constant pressure of 600 MPa during the heating. For the peak temperatures 200, 250 or 280 °C again the sample was first ramped from RT to 140 °C (13.5 K/min, dwell time 0.5 h) at 600 MPa, while during further ramping to the peak temperature (15 K/min, dwell time 1 h) the pressure was reduced to 510 MPa due to the limitations of the die mechanical stability at T > 200 °C as defined by the manufacturer.

The samples prepared using H_2_O as additive at peak temperature of 200 °C and the pressure of 510 MPa were then post-annealed at 800 °C for 1 h.

In order to perform reference experiments with conventional sintering, the LATP powder was mixed with Li_2_CO_3_ as sintering additive [14] and then uniaxially compacted with 100 MPa without heating to prepare pellets with a diameter of 8 mm. Then the pellets were pressureless heated with 3 K/min to the sintering temperatures between 760 and 840 °C for 1 h in air.

### 2.3. Characterization

After sintering, the samples were polished with sandpaper with ascending grit number changing from P600 to P2500. Then, their density was determined in ethanol based on the standard requirements described in DIN EN 1389:2003. Relative density of the samples was determined with respect to theoretical bulk density of LATP (2.94 g/cm^3^). A standard deviation of the relative density values was estimated to be of ±1.5% based on characterization of a series of eight samples, prepared using water as additive at the peak temperatures of 200 °C.

The phase composition of the materials was investigated by X-ray diffraction (XRD) in Bragg-Brentano geometry using a step scan diffractometer (Bruker D8, Bruker, Karlsruhe, Germany) with *Cu Kα* radiation (wavelength of 0.154 nm). The XRD patterns were recorded in the range of scattering angles 2*θ* = 10–90°. The XRD data were evaluated by means of the Diffrac.EVA software (Version 5.2, Bruker AXS, Karlsruhe, Germany) and crystallographic data from the ICSD- and ICDD-2021 and Pearson’s Crystal Data (2016) databases. Additionally, the lattice parameter of the samples and the phase composition was calculated using Topas 6.0 software (Bruker AXS).

In order to investigate the microstructure, the cross-sections of the cold-sintered samples were prepared by ion-beam slope cutting described in [15]. The microstructure was visualized using field emission scanning electron microscopy (FESEM, NVision40, Carl Zeiss, Oberkochen, Germany) equipped with EDX detectors. The micrographs presented in this work were acquired in the backscattered electrons mode, which allowed distinguishing between the phases with lighter and heavier elements, appearing as darker and brighter areas, respectively.

The room-temperature ionic conductivity of the sintered LATP samples was determined by electrochemical impedance spectroscopy (EIS). The polished sample surfaces were sputtered with gold to realize ion-blocking electrodes and the samples were subsequently measured in a Swagelok cell. An electrochemical workstation Zahner Zennium in combination with the Thales software (Thales XT, ZAHNER-elektrik GmbH & Co. KG, Kronach, Germany) was used for the measurements within the frequency range from 100 mHz to 1 MHz with an amplitude of 20 mV. The EIS spectra were analyzed by using RelaxIS software (RelaxIS3, rhd instruments GmbH & Co. KG, Darmstadt, Germany). An appropriate electrical equivalent circuit (EC) was fitted to the EIS data to obtain the ionic conductivity taking into account the samples’ dimensions, but without correcting the data for porosity [14]. In addition, the distribution of relaxation times (DRT) method was used to deconvolute the EIS data and to identify the electrochemical processes determining the ionic conductivity of the cold-sintered LATP. This method expresses the measured impedance *Z* by an integral equation containing the distribution function γ(τ) [16]:

(1) $$Z\left(\omega \right)=R_{0}+Z_{pol}\left(\omega \right)=R_{0}+R_{pol}\overset{\infty }{\underset{0}{\int }}\frac{\gamma \left(\tau \right)}{1+j\omega \tau }d\tau ;\overset{\infty }{\underset{0}{\int }}\gamma \left(\tau \right)d\tau =1,$$

where Z(ω) is the impedance data, $R_{0}$ is the ohmic part of the impedance, $Z_{pol}\left(\omega \right)$ is the polarization part, and $R_{pol}$ is the polarization resistance.

For the DRT calculation of the discussed EIS spectra, the low frequency polarization of the blocking electrodes was subtracted by the software. A Gaussian radial basis function (RBF) was used for discretization. The discretization factor (Lambda-factor) was set to 10^−4^. Additionally, a first-order RBF derivative and a shape factor of 0.4 was used for calculation of the DRT spectra.

Thermogravimetric analysis (TGA) of cold-sintered samples was performed in synthetic air ambience (flow 5 l/min) on a simultaneous thermal analyzer (STA 449F1, Netsch Gerätebau GmbH, Selb, Germany) in combination with emission gas mass spectroscopy (MS) by a quadrupole mass spectrometer (QMS 403 C Netsch Gerätebau GmbH, Selb, Germany). During the TGA–MS measurement, the cold-sintered samples were heated from 35 °C to 895 °C at 5 K/min and then cooled down to 20 °C at 10 K/min.

## 3. Results

### 3.1. Densification of LATP

**Figure 2:**
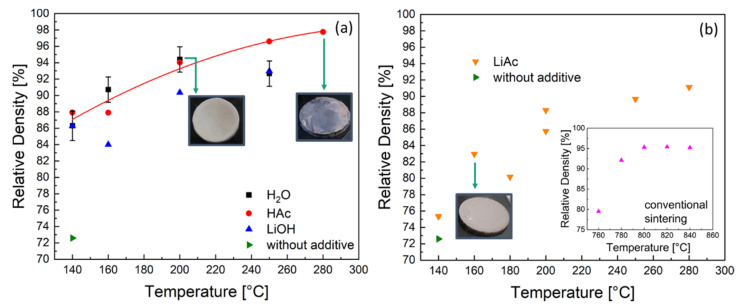
Relative density of the LATP samples prepared by cold sintering as function of the peak temperature (with 1 h dwell time) using (**a**) 20 wt.% liquid (H_2_O = water; HAc = acetic acid solution; LiOH = lithium hydroxide solution) and (**b**) 3.2 wt.% solid lithium acetate (LiAc) sintering additives. Inset in (**b**): relative density of the LATP ceramics prepared by conventional sintering at higher temperatures.

The relative density of cold-sintered LATP ceramics as function of the peak temperature is shown in Figure 2.

The cold sintering of LATP at 140 °C and 600 MPa without additives led to the material with relative density of approximately 73%, which is in agreement with literature data [12]. For the same sintering temperature and pressure with liquid sintering additives (H_2_O, HAc, LiOH) the ceramics showed higher relative densities between 86 and 88% (Figure 2a) after sintering, while in the case of solid LiAc additives, a density of only 75% was achieved (Figure 2b).

With increasing peak temperature, the densification of the material was continuously improved for all types of additives studied except the case of H_2_O (Figure 2a). Using a water additive enabled LATP ceramics with the maximum relative density of 94% at the peak temperature 200 °C. However, no further densification was possible at higher temperatures. Moreover, the samples turned out to be mechanically unstable after sintering at 250 °C and were even pulverized in case of sintering at 280 °C using H_2_O as additive. Using an HAc additive enabled LATP ceramics with approximately the same relative density of 94% as in the case of H_2_O at the same peak temperature of 200 °C. In contrast to the samples prepared using H_2_O, a further densification was observed in case of the HAc additive, yielding LATP ceramics with the highest densities of 97–98% at the peak temperatures of 250 and 280 °C.

In the case of LiOH solution, the densities of 90 and 93% were achieved at peak temperatures of 200 and 250 °C, respectively, which are approximately 5% lower compared to those of the LATP cold-sintered using H_2_O and HAc. Therefore, these LATP materials were excluded from further investigations. Using the solid LiAc additive without adding water, higher temperatures of 250 and 280 °C were required to achieve relative densities of 90%.

Conventional sintering at T = 760°C results in samples with a relative density of 80% (Figure 2b, insert). With increasing temperatures (780–840 °C) the conventionally sintered LATP ceramics are densified to 92–95%. The post-annealing for 1 h at 800 °C of the cold-sintered sample with relative density of 94% (H_2_O, 200 °C, 510 MPa) did not lead to its further densification. The resulting material exhibits a density of 95% that is consistent with the value before annealing.

### 3.2. Electrochemical Properties in Relation to the TGA–MS Results

**Figure 3:**
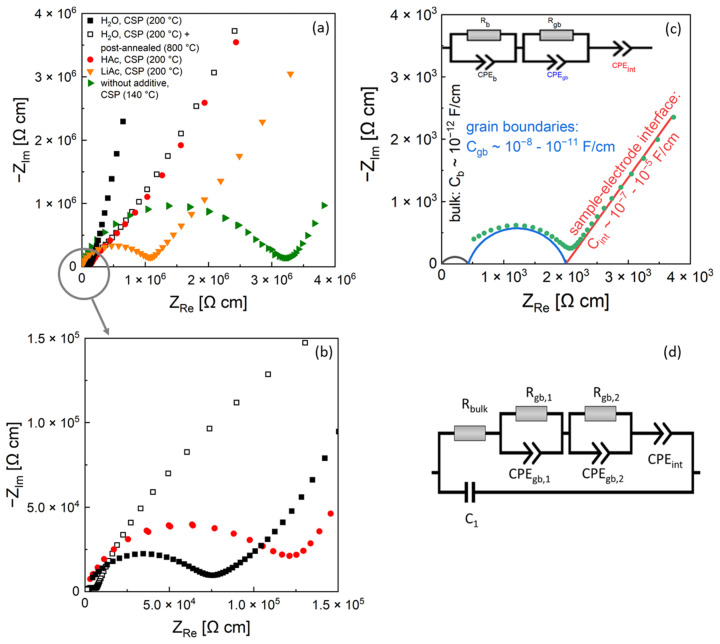
EIS spectra of cold-sintered LATP samples (**a**,**b**) and typical electrical equivalent circuit (**c**) used in literature [16] for interpretation of impedance data of ionic conducting samples with schematical illustration of the corresponding semicircles for bulk and grain boundary conductivity and straight line for blocking electrode polarization. Equivalent circuit (**d**) used in present work for fitting of EIS spectra of cold-sintered LATP samples. The EIS measurements were performed at 25 °C.

Figure 3a,b shows typical Nyquist plots of impedance spectra of LATP samples cold-sintered at the peak temperature of 200 °C using liquid (H_2_O, HAc) and solid (LiAc) additives. The data are contrasted with those of LATP ceramics cold-sintered at 140 °C without any additives and of the material post-annealed at 800 °C, which was initially cold sintered at 200 °C using H_2_O additive. Two separated elements, a semi-circle in the high frequency region and a straight line in the low frequency region [4,17], were observed in the spectra. In general, two semi-circle elements corresponding to bulk and grain boundary impedance (Figure 3c) are used in literature for analysis of EIS spectra of ion-conducting ceramics [17]. Each of these elements is represented by a resistance in parallel with a constant phase element (R–CPE), i.e., R_b_–CPE_b_ for bulk impedance and R_gb_–CPE_gb_ for grain boundary impedance in the corresponding electrical EC. To account for the capacitive charging at the interface between the LATP and ion-blocking electrode, the EC includes an additional CPE_int_ element connected in series.

The EIS data of the samples prepared by cold sintering in present work were analyzed by fitting the modified EC (Figure 3d) to the measured spectra. The best fit results as well as a summary of the corresponding fit parameter values are presented in the Supplementary Materials (Figure S1 and Table S1, respectively). As the frequency range of the semicircle element representing the bulk material conductivity lies above the frequency range of the used device, it is not directly observable in the spectra. However, the contribution of the bulk conductivity to the EIS spectra within the measured frequency range was accounted for by fitting of the simplified element represented by a single resistor R_b_. This yielded the R_b_ values within the range of 500–1000 Ω cm corresponding to ionic conductivity from 1 × 10^−3^ to 2 × 10^−3^ S/cm approaching the values of the single-crystal LATP materials [3]. In addition, the measured EIS spectra of the cold-sintered samples required two R_gb_–CPE_gb_ impedance elements in the EC to fit adequately the semi-circle feature related to the grain boundary conductivity. In contrast, conventionally sintered and post-annealed cold-sintered samples are fitted by a single R_gb_–CPE_gb_ element in the high frequency region. To account for parasitic capacity effects of the wires, a capacitor, C1 (Figure 3d), is put in parallel to the elements describing the sample and electrode processes.

The samples cold-sintered with LiAc additive at peak temperatures of 250 °C and 280 °C were not possible to fit by the suggested EC since their EIS spectra showed high noise and scattering. The ionic conductivity of the samples was roughly estimated directly from the spectra. For this purpose, the resistance value in the minimum between the high frequency semi-circle and the blocking electrode described by CPE (the straight line) is used.

**Figure 4:**
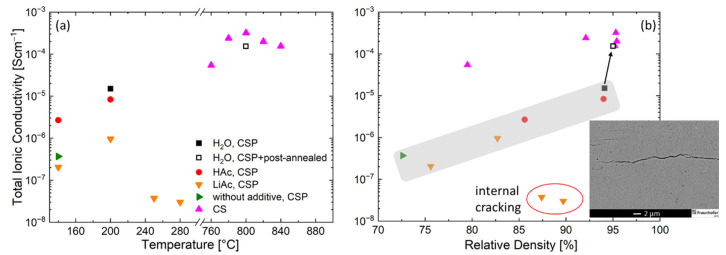
Total ionic conductivity of the LATP samples prepared by conventional pressureless sintering (CS) and cold sintering process (CSP) using liquid and solid sintering additives as a function of (**a**) the peak sintering temperature and (**b**) the relative density of the samples. Inset in (**b**): micrograph of the LATP ceramics prepared by cold sintering process at peak temperature of 280 °C using LiAc solid additive.

**Figure 5:**
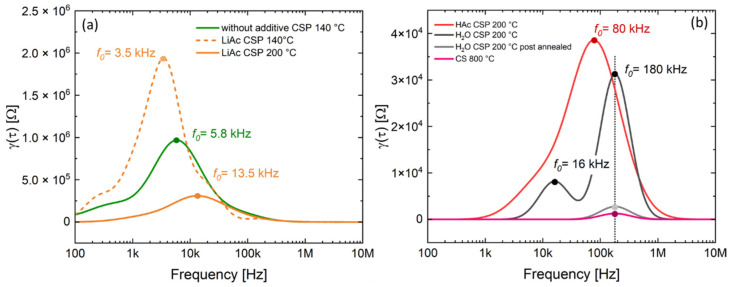
DRT spectra of cold-sintered and conventionally-sintered ceramics: (**a**) samples cold-sintered without additives at 140 °C and with LiAc at 140 and 200 °C, (**b**) samples cold-sintered with H_2_O and HAc at 200 °C as well as post-annealed and conventionally-sintered ones (both 800 °C).

Figure 4 displays the total ionic conductivity of the LATP ceramics prepared at different cold sintering temperatures as obtained from EIS measurements. The LATP material processed at 140 °C without additives indicates a low total ionic conductivity of 3.18 × 10^−7^ S/cm, which is comparable to the ionic conductivity (1.98 × 10^−7^ S/cm) of the samples with solid LiAc additive cold-sintered at the same temperature. These data are in a good agreement with DRT analysis results (Figure 5a). The dominating electrochemical processes in the samples without additives as well as in those with solid additive LiAc (Figure 5a) have low characteristic frequencies of 3.5 to 13.5 kHz and high peak resistance values within the range from 2.0 × 10^6^ to 0.3 × 10^6^ Ω, respectively. Even if prepared at higher cold- sintering temperatures of 200 °C, the sample with LiAc shows much higher resistance values compared to those with liquid additives.

Further enhancement of the sintering temperature to 250 or 280 °C using LiAc resulted in LATP ceramics with very low ionic conductivity within the range from 3 × 10^−8^ to 4 × 10^−8^ S/cm due to internal cracking (insert to the Figure 4b). This might also be the reason for the high scattering in the EIS spectra of these two samples.

Using liquid HAc additive, one order of magnitude higher ionic conductivity of 2.48 × 10^−6^ S/cm was achieved in the samples cold-sintered at 140 °C compared to those prepared at the same conditions using the solid LiAc additive. Increasing the cold sintering temperature to 200 °C led to further enhancement of the ionic conductivity with maximum values of 1.26 × 10^−5^ S/cm (H_2_O) and 0.82 × 10^−5^ S/cm (HAc). Further increase in the cold- sintering peak temperature to 250 °C in the case of H_2_O additive resulted in pulverized material that was not suitable for EIS characterization. Similarly, no reliable EIS measurement was possible also for the LATP ceramics prepared with HAc at 250 °C and 280 °C due to their very low mechanical stability and the so caused inhomogeneous surface. These samples showed strong color change from typical white to bluish one (insert Figure 2a) that is likely caused by Ti^4+^ to Ti^3+^ reduction [18,19].

The DRT data of the samples prepared at 200 °C using liquid additives (Figure 5b) show peaks at higher frequencies compared to the cases of materials cold-sintered without additives or using solid LiAc additive. In case of the sample prepared using HAc additive, a broad peak is observed at frequency of about 80 kHz. Considering the EC-fit results of the EIS spectra of the latter sample showing the characteristic frequencies of fitted R_gb_–CPE_gb_ elements at 150 kHz and 42 kHz, it may be assumed that this broad peak consists of two overlapping contributions from the processes that are not resolved in the DRT spectrum. For the sample cold-sintered using H_2_O additive at the same temperature, the dominating high-frequency peak at 180 kHz and additional smaller one at 16 kHz are observed. The peak resistance values of the latter samples are lower compared to those prepared using HAc.

Applying post-annealing of the cold-sintered sample (H_2_O, 200 °C) at 800 °C for 1 h, one order of magnitude higher ionic conductivity of 1.55 × 10^−4^ S/cm was achieved compared to the initial value of 1.26 × 10^−5^ S/cm. This is comparable to the highest values within the range from 1.55 × 10^−4^ to 3.38 × 10^−4^ S/cm of the samples resulting from conventional sintering at 780–840 °C. In both cases, applying high temperatures yields samples showing only one peak at 180 kHz in DRT spectra and displaying much lower DRT peak absolute values compared to the samples characterized directly after the cold sintering.

**Figure 6:**
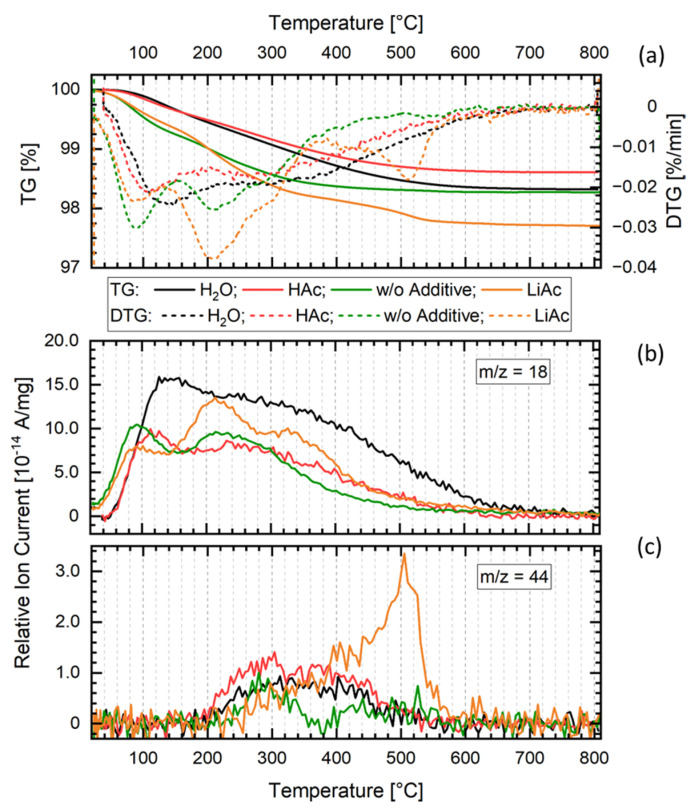
Thermogravimetry (**a**) with emission gas mass spectroscopy (**b**,**c**) (TGA–MS) of cold-sintered samples without additive and with H_2_O, Hac, and LiAc additives. *m*/*z* = 18 corresponds to water vapor and *m*/*z* = 44 to CO_2_.

Thus, the post-annealing of the cold-sintered LATP sample (H_2_O, 200 °C) leads to an increase in its total ionic conductivity by a factor of more than 10 and causes strong modification of its EIS and DRT spectra. These changes are accompanied by a total mass loss of approximately 1.5% during heating of the material to 800 °C as shown by the TGA data (Figure 6a). A similar mass loss behavior is observed for the materials cold-sintered with HAc as well as without any additives. The mass loss proceeds in two steps, the first one between 100 and 200 °C that is due to the loss of adsorbed water and the second one between 200 °C and 700 °C which is caused by the loss of crystal water from the material. MS data (Figure 6b,c) demonstrate that in the second step, apart from H_2_O, CO_2_ is emitted from the samples. The sample cold-sintered using H_2_O as additive shows a slightly higher H_2_O signal compared to the other ones, suggesting somewhat higher retention of H_2_O-containing species in that material. The low H_2_O and CO_2_ signal levels and minor differences observed among the samples do not allow further interpretation of the TGA–MS data.

TGA characterization of the LATP materials cold-sintered at 200 °C using LiAc sinter additive reveals their mass loss of about 3% (Figure 6a). It is accompanied with the CO_2_ signal in MS spectra observed for the temperatures between 400 °C and 600 °C, indicating thermal decomposition of residual LiAc (Figure 6b,c).

### 3.3. Microstructure and Phase Composition

**Figure 7:**
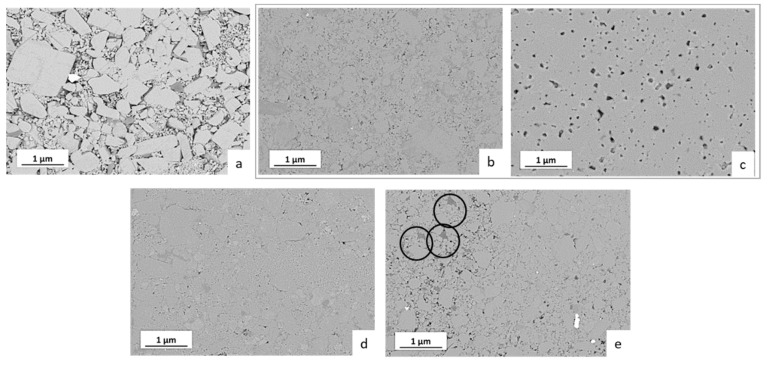
Microstructure (backscattered electrons mode) of the LATP ceramics cold-sintered at different conditions in relation to their density and ionic conductivity: (**a**) no additives, 140 °C (relative density 73%, total ionic conductivity 3.18 × 10^−7^ S/cm); (**b**) H_2_O, 200 °C (94%, 1.26 × 10^−5^ S/cm); (**c**) cold sintering using the same parameters as in (**b**) followed by post-annealing at 800 °C (95%, 1.55 × 10^−4^ S/cm); (**d**) HAc, 200 °C (94%, 0.82 × 10^−5^ S/cm); (**e**) LiAc, 200 °C (88%, 9.5 × 10^−7^ S/cm).

In order to complement the electrochemical data, the microstructure of the representative samples was investigated in more detail (Figure 7). The low-density sample prepared by cold sintering at 140 °C without additives contains separated LATP grains (Figure 7a). In contrast, the LATP samples with the highest total ionic conductivity directly after cold sintering (H_2_O or HAc additives, 200 °C) show a dense microstructure with low residual porosity (<10%) in good agreement with the measured higher density.

The LATP grains in these samples also appear to be better interconnected (Figure 7b,d), explaining higher ionic conductivities of the material. However, at the grain boundary regions of the LATP ceramics cold-sintered using the liquid additives a secondary phase seems to form, appearing in FESEM micrographs as slightly darker grey areas (Figure 7b,d). Since backscattered electrons mode of FESEM was used for recording these micrographs, it indicates enrichment in light elements in these regions compared to the LATP stoichiometry. Importantly, elimination of these areas by post-annealing at 800 °C of the cold-sintered sample (Figure 7c compared to Figure 7b) correlates with the observed improvement in the ionic conductivity of the material.

In case of LiAc solid additive, the darker-appearing areas are observable as separated grains as highlighted by the circles in Figure 7e. Similar to the case of materials cold-sintered using liquid additives, the results indicate formation of secondary phases enriched with light elements, whose presence on the grain boundary regions may also be assumed.

**Figure 8:**
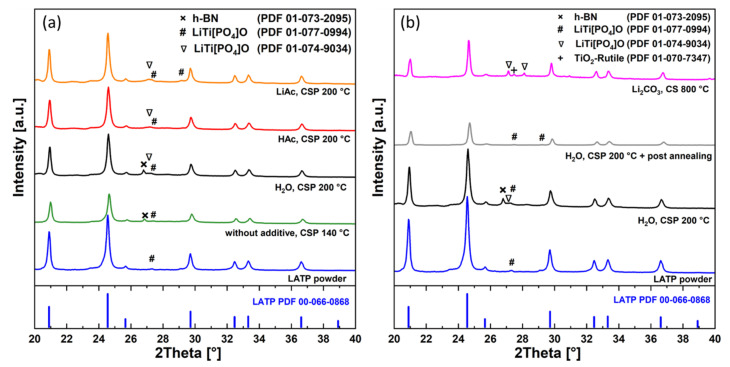
XRD patterns of the (**a**) cold-sintered samples compared with that of the as-prepared powder and (**b**) post-annealed and conventionally sintered LATP compared with as-prepared powder and cold-sintered LATP.

In addition to the microstructure characterization, the phase composition of the cold-sintered material was systematically investigated using XRD. The XRD patterns of the LATP ceramics cold-sintered without additives as well as those using H_2_O, HAc, and LiAc additives are shown in Figure 8a. They are compared with the data of the as-prepared powder, conventionally-sintered, and post-annealed LATP samples (Figure 8b). The strongest reflexes in XRD patterns of these samples are assigned to LATP (Li_1.3_Al_0.3_Ti_1.7_(PO_4_)_3_, PDF 00-066-0868). Rietveld refinement yields the LATP phase content of more than 85 wt.% for the different cold-sintered samples indicating stability of this phase under processing conditions (Supplementary Materials, Table S2). The analysis shows that the slight lattice parameters variation in cold-sintered LATP compared to the as-prepared powder is in the same range as in the case of conventionally-sintered LATP with respect to the powder (Supplementary Materials, Figure S2). This variation may indicate minor changes in the LATP stoichiometry, although no systematic trends can be detected.

A minor LiTi[PO_4_]O secondary phase is observed in all samples studied in present work as indicated by special marking of low-intensity peaks in Figure 8. The as-prepared powder is phase-pure and contains only traces of triclinic LiTi[PO_4_]O (PDF 01-077-0994). There are indications of presence of the same triclinic phase as well as of orthorhombic LiTi[PO_4_]O (PDF 01-074-9034) in all cold-sintered samples investigated (Figure 8a and, in Supplementary Materials, Figure S3 for magnified view). The LATP material initially cold-sintered (H_2_O, 200 °C) and then annealed at 800 °C also shows presence of the triclinic LiTi[PO_4_]O phase below 2 wt.%. However, in the reference sample conventionally sintered at 800 °C with addition of Li_2_CO_3_, the orthorhombic LiTi[PO_4_]O phase appears more pronounced. This phase is often reported both for cold- [20] and conventionally sintered [14,21] LATP materials.

In the reference sample (Li_2_CO_3_, 800 °C) as well as the cold-sintered ones (H_2_O, HAc, LiAc), a weak broad peak at 2*θ* ≈ 20.2° is observed. It may correspond to a nanocrystalline Li_x_TiO_2_ phase (x around 0) [22] which could be additionally stabilized by traces of Al^3+^ in the lattice. However, only this single peak could be detected in the XRD pattern for this phase. Therefore, the assignment is not unequivocal, and the peak could be also related to nanocrystalline phosphates. Due to this ambiguity, this feature is not marked in Figure 8. During annealing of the cold-sintered sample (H_2_O, 200 °C) at 800 °C, this phase disappears.

The TiO_2_ rutile secondary phase is reliably detectable only in LATP material prepared by conventional sintering (Li_2_CO_3_, 800 °C). It is also known to form in LATP sintered without additives at temperatures of 1080 °C [14].

The weak reflex at 2*θ* = 26.8° observed only in XRD patterns of the cold-sintered samples prepared without additives and those using H_2_O is attributed to the hexagonal BN (PDF 01-073-2095) from the anti-adhesion coatings (Supplementary Materials, Figure S3, magnified view). It appears to be not completely removed by the polishing procedure from the surface of these specific LATP pellets and is interpreted as a sample preparation artifact.

Thus, except the weak indications of the possible presence of nanocrystalline phases, in the present work, the cold sintering process does not induce XRD-detectable crystalline secondary phases in addition to those already observed in as-prepared LATP powder. The phases enriched in light elements observed in FESEM micrographs as darker areas could be some Li-Al-phosphates below the detection limit of XRD, or even small fractions of amorphous phases rich in light elements.

## 4. Discussion

### 4.1. Effects of Sintering Temperature and Additives on the LATP Densification

The liquid sintering additives (H_2_O, HAc, LiOH) seem to enable sufficient mass transport ensuring good densification (86–88% corresponding to 2.53–2.59 g/cm^3^) of LATP ceramics during the cold sintering process already at the peak temperature of 140 °C. It is important to note that cold sintering with the liquid additives at temperatures 140–160 °C results already in higher densification in comparison with conventional sintering at T = 760 °C. This observation agrees with the literature suggesting that cold sintering kinetics is boosted with the liquid-enhanced creep mechanism at reduced temperatures and under applied pressure [23,24].

In the case of H_2_O additive, approaching the peak temperature of 200 °C yields the material with relative density of 94% corresponding to 2.76 g/cm^3^. This value is higher than the typical cold-sintered LATP densities of 2.45–2.53 g/cm^3^ reported in the work of Lee [13] and is comparable to the values determined for the materials prepared by conventional sintering using Li_2_CO_3_ as additive at 800 °C in the present work (2.79 g/cm^3^) and at 775 °C (2.78 g/cm^3^) in the work of Xu [21].

In the specific case of H_2_O additive, increasing the cold sintering temperature above the optimum value of 200 °C leads to mechanically unstable material. It may be speculated that the most likely reason for the observed behavior is too fast removal of water from the sample at temperatures of 250 and 280 °C so that sufficient mass transport can no longer be ensured.

Since the HAc additive was used in the form of water solution, water is also expected to be removed from the sample in the same way as in the previous case. However, the equilibrium pressure is reduced as the solution becomes more concentrated in HAc. Additionally, remaining residuals of HAc and reaction products (acetates) most likely enable sufficient mass transport at these temperatures and promote the observed densification to the values of 97–98% (2.85–2.88 g/cm^3^). The latter assumption is supported by experiments with solid LiAc additive (Figure 2b) showing substantial material densification at the temperatures of 250 and 280 °C that are in the range of the melting point of water-free LiAc.

Neither conventional sintering at even higher temperatures (780–840 °C) using Li_2_CO_3_ additive, nor post-annealing at 800 °C, is observed to improve the relative density above the 94% determined in optimized cold sintering samples.

### 4.2. Ionic Conductivity in Relation to the Density and Microstructure of the Cold-Sintered LATP

Understanding factors limiting ionic conductivity of the cold-sintered LATP is necessary for further improvement in its properties to make it suitable for solid-state battery applications. The highest ionic conductivity value achieved in this work (1.26 × 10^−5^ S/cm, H_2_O, 200 °C) is of the same order of magnitude as reported in literature for cold-sintered LATP (2.8 × 10^−5^ S/cm), where water with NMP was used as an additive and cold sintering at 120 °C was combined with post-annealing at 650 °C [12]. The ionic conductivity increases with relative density both for cold- and conventionally sintered LATP, but the cold-sintered samples having higher relative density show much lower ionic conductivity than conventionally sintered ones (Figure 4b). Therefore, achieving high relative density is not sufficient to ensure high ionic conductivity of the LATP and EIS data should be related to the microstructure and phase composition of the material.

Based on the EIS (Figure 3) and DRT results (Figure 5), one may assume that in the cold-sintered sample (H_2_O, 200 °C), initially two different kinds of grain boundaries are formed, which are represented by two DRT peaks at 16 and 180 kHz. The minor crystalline secondary phase LiTi[PO_4_]O detected by XRD (Figure 8) occurs in a form of separated grains appearing as slightly brighter areas in SEM and not affecting the grain boundaries [21]; therefore, it is not related to these DRT features. Moreover, this secondary phase is observed even in conventionally sintered LATP materials with high ionic conductivity similar to the results of [21] and, hence, it is not expected to affect Li-ion conduction pathways substantially. On the other hand, it can be speculated that these types of grain boundaries are related to the minor formation of disordered/amorphous phases enriched in light elements, which are observed as darker areas in FESEM micrographs (Figure 7b,d). They are supposed to hamper Li-ion conduction pathways in cold-sintered LATP ceramics and cause their reduced ionic conductivity compared to conventionally sintered ones. Formation of such amorphous/glassy phases at the grain boundaries is known for different cold-sintered materials [11]. In the case of LATP, this may be attributed to its incongruent dissolution in water under cold sintering conditions starting with Li removal and triggering the secondary phase formation [25]. However, extensive structural investigations are necessary to define the exact pathway of the secondary phase formation under our specific experimental conditions.

The assumption that the amorphous/disordered secondary phases at the grain boundaries limit ionic conduction of the cold-sintered LATP is further confirmed by post-annealing at 800 °C of the initially cold-sintered (H_2_O, 200 °C) material. This treatment enhances total ionic conductivity of the LATP by approximately one order of magnitude (from 1.26 × 10^−5^ to 1.55 × 10^−4^ S/cm) without observable changes in relative densities, suggesting improvement in contact among the grains most likely due to thermal decomposition and transformation of the secondary phases at the grain boundaries. This agrees with the observed elimination of the DRT peak at 16 kHz and strong decrease in the amplitude of the 180 kHz peak of the thermally annealed sample. Since the peak at 180 kHz persists after the heat treatment with much lower peak amplitude, it can be implied that the corresponding type of the grain boundaries forms already during the cold sintering, but their thickness decreases compared to the initial cold-sintered sample. This is in agreement with literature data [21,26], showing existence of amorphous or highly disordered phases in grain boundary regions with a thickness of a few nm even in the conventionally sintered LATP. Finally, FESEM images confirm the decomposition of the disordered phases enriched with lighter elements during post-annealing step (Figure 7c) showing no darker areas observed in the initially cold-sintered material (Figure 7b).

In contrast to the LATP ceramics prepared with H_2_O, the EIS and DRT results of the LATP cold-sintered at 200 °C using solid LiAc sintering additive indicate that nearly no conductive grain boundaries are formed in this case, or the grains are separated by residual LiAc after the sintering process. This assumption is supported by an observed mass loss of about 3% and CO_2_ signal between 400 °C and 600 °C in the TG–MS data (Figure 6a,c) indicating thermal decomposition of the residual LiAc. Like in the case of H_2_O additive, the minor formation of other amorphous or disordered phases in the grain boundary regions may also play a role in decreasing ionic conductivity of this material.

Formation of microcracks appears to be another factor limiting ionic conductivity in the samples cold-sintered with solid LiAc additive at 250 and 280 °C (Figure 4b, insert). It is likely that during cooling down and depressurization, the internal stress cannot be relieved without any liquid phase, resulting in the observed cracking.

The present work indicates the importance of blocking or minimizing amorphous/disordered phase formation at the grain boundaries during cold sintering of LATP to ensure its high ionic conductivity. Based on the results of Hamao [20], one may assume that high-temperature treatment of LATP tape during debindering at 650 °C for 2 h prior to the cold sintering modifies the grain-to-grain contact regions in the initial material compared to the case of LATP mass usually used for the cold sintering. This seems to prevent formation of the undesirable phases in cold-sintered LATP tape and to enable total ionic conductivity in this material as high as 3 × 10^−4^ S/cm directly after cold sintering (0.5 h, H_2_O, 200 °C, 25 MPa). Although the authors did not discuss this effect in [20], it should be investigated further along with experimental efforts to limit incongruent dissolution of LATP and to understand the possibility of achieving ionic conductivities as high as 10^−4^ S/cm by cold sintering without high temperature pre- or post-treatment.

## 5. Conclusions

Highly dense LATP ceramics with relative densities >90% of the LATP theoretical value were realized for liquid (H_2_O, HAc, LiOH) and solid (LiAc) additives at temperatures below 280 °C and pressure of 510–600 MPa. With a relative density of 94%, the total ionic conductivity as high as 1.26 × 10^−5^ and 0.82 × 10^−5^ S/cm was obtained at the optimum sintering temperature of 200 °C using H_2_O and aqueous solution of HAc as sintering additives, respectively.

In case of liquid additives such as H_2_O and HAc, highly densified microstructures with minor closed porosity were observed after cold sintering. The sintering additives seem to partially induce formation of the small amounts of amorphous phases in the grain boundary regions. The formation of these secondary phases is supposed to limit the ionic conductivity of the cold-sintered material. This assumption is supported by analysis of the EIS data with appropriate EC and DRT deconvolution showing contributions related to two different grain boundary types. Furthermore, post-treatment at 800 °C resulted in decomposition or transformation of these phases, leading to one order of magnitude higher ionic conductivity correlating to a single grain boundary type contribution both in EC and DRT spectra.

Similar processes limiting ionic conductivity were observed in the cold-sintered samples prepared using solid additive LiAc. Additionally, indications of residual LiAc were detected by TGA–MS in the material prepared at 200 °C. In contrast to the materials prepared using liquid additives, formation of microcracks appears to be another factor limiting ionic conductivity in the samples cold-sintered with solid LiAc additive.

These results suggest applicability of the cold sintering for preparing solid Li-ion conducting ceramics as electrolyte for all solid-state batteries. The demonstrated temperature range is suitable for co-sintering of electrolyte and cathode materials that will be investigated further. Especially, the approaches for improvement in the ionic conductivity of the cold-sintered LATP material should be developed further, focusing on removing the factors deteriorating ionic transport. The detailed mechanisms of the secondary phase formation in the grain boundary regions of LATP should be investigated to minimize their effect on the ionic conductivity of the material.

## Figures and Tables

**Table 1:** Summary of the sintering parameters.

Mode	Additives	Heating Profile (Ramp (K/min), Constant Temperature (°C), Dwell Time (h)) and Maximum Pressure (MPa)
Cold sintering	No additives, H_2_O, HAc, LiOH, LiAc	13.5 K/min from RT to 140 °C (1 h/600 MPa)
Cold sintering	H_2_O, HAc, LiOH, LiAc	13.5 K/min from RT to 140 °C (0.5 h/600 MPa) → 15 K/min to 160 or 180 °C (1 h/600 MPa)
Cold sintering	H_2_O, HAc, LiOH, LiAc	13.5 K/min from RT to 140 °C (0.5 h/600 MPa) → 15 K/min to 200, 250 or 280 °C (1 h/510 MPa)
Conventional sintering	Li_2_CO_3_	13.5 K/min RT to 760, 780, 800, 820 or 840 °C (1 h)

## Data Availability

The data presented in this study are available on request from the corresponding authors.

## Supplementary Materials

The following supporting information can be downloaded at: https://www.mdpi.com/article/10.3390/nano12183178/s1. Figure S1: Best fit results using equivalent circuit (Figure 3c) shown for different cold-sintered LATP samples in comparison to the post-annealed one. Overview of the impedance spectra analyzed in present work (left) along with magnified view (right) of the samples with higher ionic conductivity is presented. Figure S2: Lattice parameters *a* (a) and *c* (b) as well as the unit cell volume (c) of the LATP materials prepared using different methods. The notation “powder” corresponds to as-prepared LATP powder material in present work. CS stands for the LATP material prepared using conventional sintering: “CS 1080 °C” corresponds to sintering temperature of 1080 °C without additives as in Ref. [14], “CS Li2CO3 800” means conventional sintering of LATP using Li_2_CO_3_ as an additive at sintering temperature of 800 °C. CSP means “cold sintering process”; the XRD data are shown then for different sintering additives (H_2_O, HAc, LiAc) and the case where no additives were used (“w/o”); the temperature of CSP is also shown (140 or 200 °C). Figure S3: The magnified view of the XRD patterns of the cold-sintered samples compared with that of the as-prepared powder (left) and post-annealed and conventionally sintered LATP compared with as-prepared powder and cold-sintered LATP (middle). The right-hand side image shows further magnified XRD pattern from the middle image suggesting that even the weakest peak related to the LiTi[PO_4_]O phase is detectable. Table S1: Best-fit parameters of the corresponding equivalent circuit (Figure 3c) of the LATP samples cold-sintered at different conditions in comparison to those of the post-annealed material; Table S2: Content of the LATP phase (PDF 00-066-0868) in LATP materials prepared at different conditions as derived from the Rietveld analysis.
