# Supplementary material for: Li-Ion Conductive Li1.3Al0.3Ti1.7(PO4)3 (LATP) Solid Electrolyte Prepared by Cold Sintering Process with Various Sintering Additives
Source: Nanomaterials (Basel). 2022 Sep 13;12(18):3178. doi: 10.3390/nano12183178 (PMC9503772; doi:10.3390/nano12183178)
Supplement: Supplementary file 1 [file nanomaterials-12-03178-s001.zip › nanomaterials-1804893-supplementary.pdf]

Supplementary Materials

The fitting results are shown in the Figure S1. The raw EIS data are presented as the points. The same data sets are used as in the Figure 3a,b. The best fit results are shown as the lines of the corresponding color. Very low frequency data, which were not assigned to the sample itself or the blocking electrode, were removed from the spectra before fitting. Exact numerical values of the fit parameters for all fitted spectra are also shown in the Table S1 below. The R-CPE element with Rgb2 and CPE gb2 of the sample cold sintered at 140 °C without additive is excluded from conductivity measurement. Due to its low characteristic frequency in the region of 10 Hz this process is assigned to electrode polarization effects and not to ionic conductivity in the LATP electrolyte material.

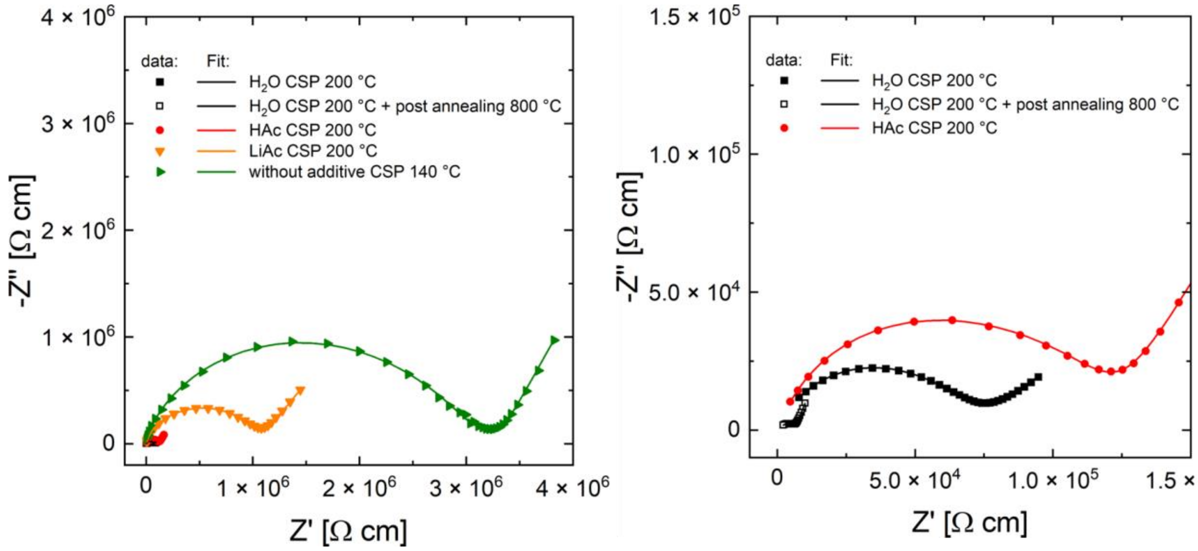

Figure S1. Best fit results using equivalent circuit (Figure 3c) shown for different cold-sintered LATP samples in comparison to the post-annealed one. Overview of the impedance spectra analyzed in present work (left) along with magnified view (right) of the samples with higher ionic conductivity is presented.

Table S1. Best fit parameters of the corresponding equivalent circuit (Figure 3c) of the LATP samples cold sintered at different conditions in comparison to those of the post-annealed material.

**Additive**		**w/o additive**	**LiAc**	**LiAc**	**HAc**	**HAc**	**H_2O**	**H_2O**	**CS**	**CS**	**CS**	**CS**	**CS**
T (°C)		140	140	200	140	200	200	200–800	760	780	800	820	840
R_{bulk}	[Ω cm]	0.00	0.00	0.00	0.00	727.26	800.00	802.24	1378.17	578.95	466.08	446.18	471.25
$R_{gb,1}$	[Ω cm]	3,140,957.19	491,392.02	119,120.14	32,698.26	36,791.11	56,432.02	5670.17	16,412.39	3758.61	2495.53	4639.06	5993.44
$CPE_{gb.1}$ Q	[F cm^{-1} s$^{\alpha-1}$]	5.62×10^{-10}	9.14×10^{-11}	3.90×10^{-11}	3.23×10^{-11}	1.94×10^{-10}	1.03×10^{-9}	1.75×10^{-9}	8.63×10^{-10}	3.35×10^{-9}	3.81×10^{-9}	3.25×10^{-9}	2.77×10^{-9}
$CPE_{gb.1}$ α		0.59	0.84	0.93	1.00	0.86	0.69	0.78	0.81	0.76	0.79	0.82	0.81
$R_{gb.2}$	[Ω cm]	150,468.50	4,560,207.88	929,918.71	370,069.10	84,892.92	22,424.55	-	-	-	-	-	-
$CPE_{gb.2}$ Q	[F cm^{-1} s$^{\alpha-1}$]	2.34×10^{-7}	2.10×10^{-10}	5.03×10^{-10}	2.07×10^{-9}	2.20×10^{-9}	4.14×10^{-7}	-	-	-	-	-	-
$CPE_{gb.2}$ α		0.77	0.70	0.68	0.59	0.69	0.41	-	-	-	-	-	-
CPE_{int} Q	[F cm^{-1} s$^{\alpha-1}$]	1.55×10^{-6}	1.65×10^{-7}	1.35×10^{-7}	5.20×10^{-7}	1.29×10^{-7}	1.40×10^{-6}	4.25×10^{-8}	2.56×10^{-6}	9.83×10^{-8}	4.07×10^{-6}	2.04×10^{-6}	2.46×10^{-6}
CPE_{int} α		0.68	0.59	0.57	0.50	0.67	0.50	0.77	0.59	0.83	0.60	0.64	0.65
C_1	[F cm^{-1}]	2.63×10^{-12}	2.64×10^{-12}	1.94×10^{-12}	3.27×10^{-12}	2.00×10^{-12}	2.74×10^{-12}	2.00×10^{-12}	2.00×10^{-12}	2.00×10^{-12}	2.00×10^{-12}	2.00×10^{-12}	2.00×10^{-12}

Table S2. Content of the LATP phase (PDF 00-066-0868) in LATP materials prepared at different conditions as derived from the Rietveld analysis.

Preparation conditions	LATP phase content (wt.%)
As-prepared powder	98.2
Conventional sintering. no additives. 1080 °C [14]	91.9
Conventional sintering. Li_2CO_3. 800 °C	81.7
Cold sintering process. H_2O. 200 °C	90.9
Cold sintering process. H_2O. 200 °C followed by annealing at 800 °C	98.4
Cold sintering process. HAc. 200 °C	88.1
Cold sintering process. LiAc. 200 °C	85.5
Cold sintering process. without additives. 140 °C	94.8

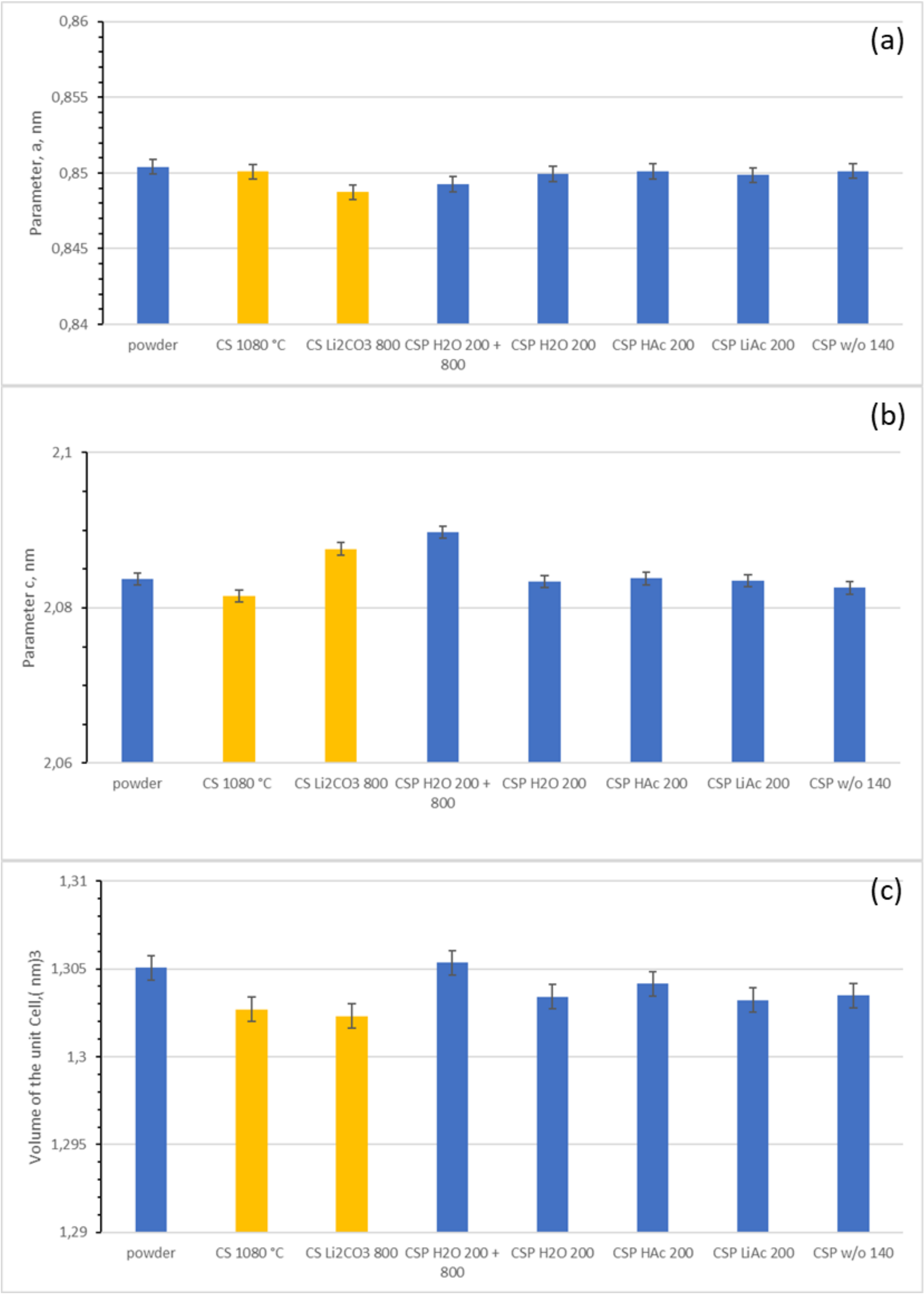

Figure S2. Lattice parameters *a* (**a**) and *c* (**b**) as well as the unit cell volume (**c**) of the LATP materials prepared using different methods. The notation "powder" corresponds to as-prepared LATP powder material in present work. CS stands for the LATP material prepared using conventional sintering: "CS 1080 °C" corresponds to sintering temperature of 1080 °C without additives as in Ref. [14]. "CS Li2CO3 800" means conventional sintering of LATP using Li_2CO_3 as an additive at sintering temperature of 800 °C. CSP means "cold sintering process". the XRD data are shown then for different sintering additives (H2O. HAc. LiAc) and the case where no additives were used ("w/o"). the temperature of CSP is also shown (140 or 200 °C).

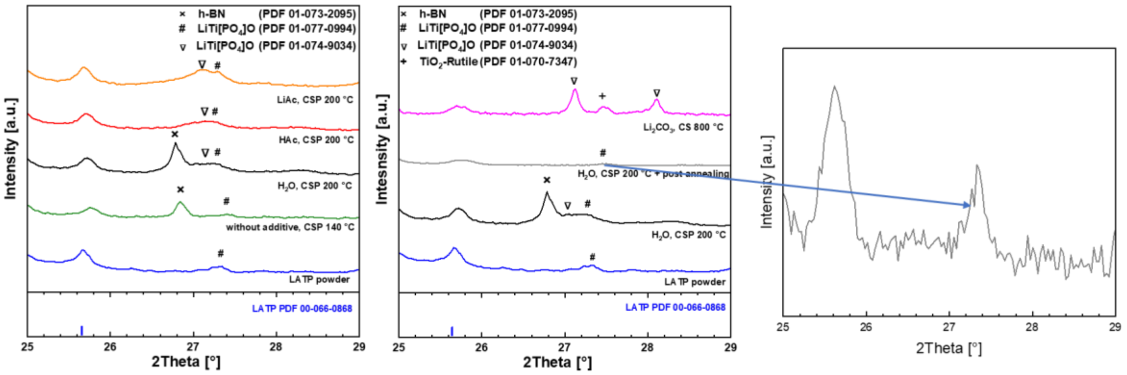

Figure S3. The magnified view of the XRD patterns of the cold-sintered samples compared with that of the as-prepared powder (left) and post-annealed and conventionally sintered LATP compared with as-prepared powder and cold-sintered LATP (middle). The right-hand side image shows further magnified XRD pattern from the middle image suggesting. that even the weakest peak related to the LiTi[PO$_4$]O phase is detectable.
